# Comparative Prevalence of Cerebrovascular Disease in Vietnamese Communities in South-Western Sydney

**DOI:** 10.3390/jcdd11060164

**Published:** 2024-05-24

**Authors:** Deena Alysha, Christopher Blair, Peter Thomas, Timmy Pham, Tram Nguyen, Theodore Ross Cordato, Helen Badge, Nicola Chappelow, Longting Lin, Leon Edwards, James Thomas, Suzanne Hodgkinson, Cecilia Cappelen-Smith, Alan McDougall, Dennis John Cordato, Mark Parsons

**Affiliations:** 1 Department of Neurology and Neurophysiology, Liverpool Hospital, Sydney, NSW 2170, Australia; z5282227@ad.unsw.edu.au (D.A.); christopher.blair@health.nsw.gov.au (C.B.); timmy.pham@health.nsw.gov.au (T.P.); tram.nguyen@health.nsw.gov.au (T.N.); rcordato99@gmail.com (T.R.C.); nicola.chappelow@health.nsw.gov.au (N.C.); james.thomas2@health.nsw.gov.au (J.T.); s.hodgkinson@unsw.edu.au (S.H.); mark.parsons@unsw.edu.au (M.P.); 2 Ingham Institute for Applied Medical Research, Sydney, NSW 2170, Australia; longting.lin@unsw.edu.au; 3 South-Western Sydney Clinical School, University of New South Wales, Sydney, NSW 2170, Australia

**Keywords:** stroke, transient ischaemic attack, cardiovascular risk factors, culturally and linguistically diverse communities

## Abstract

Culturally and linguistically diverse (CALD) communities are growing globally. Understanding patterns of cerebrovascular disease in these communities may improve health outcomes. We aimed to compare the rates of transient ischaemic attack (TIA), ischaemic stroke (IS), intracerebral haemorrhage (ICH), intracranial atherosclerosis (ICAD), and stroke risk factors in Vietnamese-born residents of South-Western Sydney (SWS) with those of an Australian-born cohort. A 10-year retrospective analysis (2011–2020) was performed using data extracted from the Health Information Exchange database characterising stroke presentations and risk factor profiles. The rates of hypertension (83.7% vs. 70.3%, *p* < 0.001) and dyslipidaemia (81.0% vs. 68.2%, *p* < 0.001) were significantly higher in Vietnamese patients, while the rates of ischaemic heart disease (10.4% vs. 20.3%, *p* < 0.001), smoking (24.4% vs. 40.8%, *p* < 0.001), and alcohol abuse (>1 drink/day) (9.6% vs. 15.9%, *p* < 0.001) were lower. The rates of ICAD and ICH were higher in Vietnamese patients (30.9% vs. 6.9%, *p* < 0.001 and 24.7% vs. 14.4%, *p* = 0.002). Regression analysis revealed that diabetes (OR: 1.86; 95% CI: 1.14–3.04, *p* = 0.014) and glycosylated haemoglobin (OR: 1.51; 95% CI: 1.15–1.98, *p* = 0.003) were predictors of ICAD in Vietnamese patients. Vietnamese patients had higher rates of symptomatic ICAD and ICH, with unique risk factor profiles. Culturally specific interventions arising from these findings may more effectively reduce the community burden of disease.

## 1. Introduction

Culturally and linguistically diverse (CALD) communities have unique demands for health service delivery and provision [1]. CALD communities, both in Australia and globally, are at increased risk of health inequities and adverse health-related outcomes [2]. Contributing factors may include unfamiliarity with health services and incomplete understanding of health information [3,4]. Traditional methods of healthcare may also conflict with evidence-based recommendations for primary and secondary prevention [4,5].

The Vietnamese-born population in Australia has grown by 32.8% in the past decade to 281,810 people, as of June 2022, constituting the country’s sixth largest migrant community and equating to 1.1% of Australia’s total population [6]. This compares to a population of 700 fifty years ago who were mostly comprised of tertiary students, orphans, and wives of Australian military personnel who served in Vietnam [6]. In part, due to a relatively recent influx of migration into Australia beginning in the late 1970s, the median age of Vietnamese-born Australians is 48.1 years, 9.6 years greater than that of the general population, with a female-to-male ratio of 1.28:1 [6].

South-Western Sydney (SWS) accounts for 17% of the population of New South Wales (NSW), Australia’s most populated state, of which 47% are born overseas, making it one of the most culturally and linguistically diverse regions of Australia. In the 2021 Australian Census, 40,534 SWS residents were born in Vietnam, accounting for 41% of NSW’s Vietnamese-born residents. In general, the same Census also reported that SWS had higher rates of unemployment, lower household income, and lower educational attainment levels compared to both state and national averages [7]. A demographic study reported that 68% of households predominantly speak a non-English language. Vietnamese was the most common language spoken (11.4%), with 8.5% of respondents born in Vietnam [8].

A recent SWS study compared stroke characteristics in Pacific Islander communities to an unselected Australian cohort, revealing that Pacific Islander patients had substantially higher rates of hypertension, atrial fibrillation (AF), diabetes, chronic kidney disease, obesity, smoking, and excess alcohol intake [9]. Certain stroke types were more common and, crucially, this preponderance of disease correlated with potentially modifiable risk factor exposure in a manner not seen in control participants. For example, the rates of intracranial atherosclerotic disease (ICAD) in age- and sex-matched Indo-Fijian patients were more than double those seen in both Indigenous Fijians (geographically matched, ethnically nonmatched) and the controls (geographically and ethnically nonmatched). Furthermore, Indo-Fijian heritage was an independent predictor of ICAD that uniquely interacted with insulin-dependent diabetes, identifying this as a priority target for risk factor modification [9].

Improved understanding of risk factor prevalence and patterns of disease amongst CALD communities facilitates targeted strategies for culturally appropriate healthcare and creates opportunities to address health inequities with more effective resource allocation [10,11,12]. To facilitate culturally competent health service provision, we examined the cerebrovascular disease characteristics and rates of transient ischaemic attack (TIA) and ischaemic and haemorrhagic stroke in Vietnamese-born patients presenting to a large stroke centre within the SWS Local Health District (LHD) and compared the trends identified with those seen in an Australian-born cohort.

## 2. Materials and Methods

This was a retrospective study for the 10-year period of January 2011 to December 2020. Data for SWS were extracted from the Health Information Exchange (HIE) of South-Western Sydney Local Health District (SWSLHD). The patients included in this study were first-generation Vietnamese-born individuals. Only the first admission of the person by year during this time period was included in this study. The data were extracted based on discharge TIA, ischaemic stroke (IS), or intracranial cerebral haemorrhage (ICH) diagnoses. Transient ischaemic attack, IS, and ICH were defined in accordance with ICD-10-AM (Australian modification) codes and the National Stroke Foundation, Australia.

The demographic data collected included the following: admission date, sex, age, estimated stroke onset date and time, admission/premorbid Modified Rankin Scale (mRS), arrival to ED via ambulance (versus private transport), body mass index (BMI), the presence of prior TIA/stroke or ischaemic heart disease, presence of AF (newly diagnosed, pre-existing, undertreated/not treated), prior hypertension (admitted on antihypertensives and how many), dyslipidaemia (including cholesterol at admission, and admitted on lipid-lowering therapy), presence of prior diabetes (divided into non-insulin-dependent and insulin-dependent), and current glycosylated haemoglobin (HbA1c earliest from admission); history of current or past smoking, history of excessive alcohol consumption and presence of chronic kidney disease (determined by highest estimated glomerular filtration rate (eGFR) during admission), type of presentation (TIA, ischaemic stroke, or intracerebral haemorrhage), imaging characteristics, receipt of hyperacute treatment (tPA, EVT, or both), strokes Oxfordshire Classification, stroke subtype according to the TOAST classification, discharge mRS, and in-hospital death. ICAD was classified as more than 50% stenosis or “moderate” to “severe” stenosis on CT angiogram or MRI/MRA. Data were collected from a medical chart review by co-authors DA, RC, and TP and verified by senior co-authors DC, CB, and HB. The direct age-standardisation method was used for the age-standardised calculations. Unadjusted and age-standardised rates per 100,000 population/year of TIA, IS, and ICH and the five stroke risk factors were determined for each group.

The study received ethics approval from South-Western Sydney Local Health District Human Research Ethics Committee (2019/ETH09989). All the data analyses were performed with SPSS software (Version 23 for Windows, SPSS, IBM Corp., Armonk, NY, USA).

## 3. Results

The Vietnamese cohort consisted of 405 patients; 54 (17.7%) patients presented with TIA, 251 (62.0%) with ischaemic stroke, and 100 (24.7%) with haemorrhagic stroke (Table 1). Of the 251 Vietnamese patients with an ischaemic stroke, 59 (23.5%) had a previous stroke. Of the 100 Vietnamese patients with a haemorrhagic stroke, 23 (23.0%) had a previous stroke. Of the total Vietnamese cohort including TIA presentations, 93 (23.0%) had a previous stroke.

BMI was found to be significantly lower in Vietnamese patients compared to control participants (Table 2). A total of 30 (11.8%) Vietnamese patients received acute stroke intervention compared to 86 (25.7%) Caucasian patients; 10 (3.9%) Vietnamese patients received thrombolysis alone, 11 (4.3%) received endovascular thrombectomy (EVT) alone, and 9 (3.5%) received both thrombolysis and EVT. In contrast, among Caucasian patients, 30 (9.0%) received thrombolysis alone, 31 (9.3%) received EVT alone, and 25 (7.5%) received both (Table 1). There were 84 (20.7%) all-case in-hospital deaths in Vietnamese patients and 63 (16.2%) in Caucasians.

### 3.1. Stroke Risk Factors and Subtypes

The rates of hypertension (83.7% vs. 70.3%, *p* < 0.001) and dyslipidaemia (81.0% vs. 68.2%, *p* < 0.001) were significantly higher in Vietnamese compared to Caucasian patients (Table 2). In contrast, the incidences of IHD (20.3% vs. 10.4%, *p* < 0.001), smoking (40.8% vs. 24.4%, *p* < 0.001), and alcohol abuse (>1 drink/day) (15.9% vs. 9.6%, *p* < 0.001) were significantly higher in Caucasians compared to Vietnamese patients (Table 2). The rates of ICAD and ICH were significantly higher in Vietnamese patients compared to Caucasian patients (30.9% vs. 6.9%, *p* < 0.001 and 24.7% vs. 14.4%, *p* = 0.002, respectively) (Table 1).

### 3.2. Predictors of Disease: Intracranial Atherosclerotic Disease (ICAD)

Multiple regression analysis revealed that Vietnamese ethnicity (OR 4.29; 95% CI 2.65–6.96, *p* < 0.001), HbA1c (OR 1.37; 95% CI: 1.12–1.66, *p* = 0.002), and diabetes (OR 1.56; 95% CI 1.03–2.37, *p* = 0.036) were associated with ICAD in the entire cohort. The subgroup analysis of Vietnamese patients revealed that diabetes (OR: 1.86; 95% CI: 1.14–3.04, *p* = 0.014) and HbA1c (OR: 1.51; 95% CI: 1.15–1.98, *p* = 0.003) were independent predictors of ICAD. No significant findings were found in the Caucasian subgroup analysis, suggesting that these factors may not play the same role in ICAD risk among Caucasians. Ethnicity was also an independent predictor of symptomatic ICAD (OR: 6.47; 95% CI: 3.39–12.37, *p* < 0.001).

### 3.3. Predictors of Disease: Intracerebral Haemorrhage (ICH)

Multiple regression analysis of the entire cohort revealed that Vietnamese ethnicity (OR: 2.26; 95% CI: 1.47–3.46, *p* < 0.001) and statin exposure (OR: 3.72; 95% CI: 1.69–8.20, *p* = 0.001) were independent predictors of ICH. In contrast to Caucasians, being prescribed statins was found to be an independent predictor of ICH in subgroup analyses of the Vietnamese cohort (OR: 3.87; 95% CI: 1.54–9.68, *p* = 0.004).

### 3.4. Predictors of Disease: Total Anterior Circulation Infarct (TACI)

Multiple regression analysis revealed that Vietnamese ethnicity (OR: 3.91; 95% CI: 1.58–9.67, *p* = 0.001), smoking (OR: 3.54; 95% CI: 1.42–8.84, *p* = 0.007), and IHD (OR: 5.46; 95% CI: 2.25–13.22, *p* < 0.001) were independent predictors of TACI. IHD was found to be an independent predictor of TACI in the subgroup analysis of the Vietnamese cohort (OR: 5.10; 95% CI: 1.58–16.45, *p* = 0.006) and the Caucasian cohort (OR: 6.94; 95% CI: 1.51–31.87, *p* = 0.013).

### 3.5. Predictors of Disease: Cardioembolic Stroke

Multiple regression analysis revealed that Caucasian ethnicity (2.01; 95% CI: 1.12–3.60, *p* = 0.019), AF (OR: 39.42; 95% CI: 21.77–71.38, *p* < 0.001), IHD (OR: 2.01; 95% CI: 1.01–4.00, *p* = 0.046), and BMI (OR: 1.06; 95% CI: 1.01–1.12, *p* = 0.030) were independent predictors of cardioembolic stroke for the whole cohort. In the Vietnamese subgroup only, BMI (OR: 1.06; 95% CI: 1.01–1.12, *p* = 0.030) and IHD (OR: 6.44; 95% CI: 2.00–20.68, *p* = 0.002) were found to be independent predictors of cardioembolic stroke.

## 4. Discussion

This study revealed notable disparities between Vietnamese and Caucasian stroke patients. Vietnamese patients had elevated rates of hypertension and dyslipidaemia with lower BMIs, while Caucasians had higher rates of ischemic heart disease, smoking, and alcohol use. Vietnamese patients were more prone to intracranial atherosclerotic disease (ICAD) and intracerebral haemorrhage (ICH), with ethnicity being a significant predictor for both stroke aetiologies. Vietnamese ethnicity, smoking, and ischemic heart disease (IHD) emerged as independent predictors for total anterior circulation infarct (TACI). Notably, ischemic heart disease was a significant predictor of TACI in both Vietnamese and Caucasian cohorts. In the study, the Oxfordshire Community Stroke Project Classification was used to categorise different stroke subtypes. This classification system helps identify specific patterns of stroke presentation. Identifying a higher prevalence of certain subtypes like TACI among Vietnamese patients highlights the importance of addressing specific risk factors such as smoking and ischemic heart disease in stroke prevention strategies targeted at this community.

Caucasian ethnicity, AF, IHD, and BMI were predictors of cardioembolic stroke, with BMI and IHD being linked to cardioembolic stroke in the Vietnamese subgroup. Patients of Vietnamese background exhibited a higher in-hospital mortality rate and received acute stroke interventions less frequently. The Vietnamese cohort consisted of Vietnamese-born individuals, for whom the net health effects of social integration would be less pronounced than for second- and third-generation immigrants.

### 4.1. BMI/Hypertension/Dyslipidaemia

Vietnamese patients had significantly lower BMIs, a trend consistent with prior findings among newly arrived Vietnamese refugees in Australia [13]. This discrepancy may be attributed partly to cultural and dietary differences, emphasizing the influence of acculturation on health behaviours [14]. Moreover, while smoking rates in Vietnam are relatively high, the current study observed a higher prevalence of smoking among Caucasians, potentially implicating the influence of acculturation on lifestyle changes. Hypertension, a well-established stroke risk factor [15], was observed at higher rates in a US-based study, with Filipino and Vietnamese individuals more likely to have hypertension compared to other ethnic minority groups [16]. Hypertension is also a significant public health concern among Vietnamese adults domestically, being the third leading contributor to cause of death in Vietnamese hospitals among adults [17]. Dyslipidaemia was also more prevalent among Vietnamese patients. This observation aligns with the findings of higher rates in certain Asian American subgroups in a comparable study, with Vietnamese participants found to have the second highest rates of dyslipidaemia among diverse racial/ethnic groups in the US [18].

### 4.2. ICAD

Intracranial atherosclerotic disease (ICAD) and intracerebral haemorrhage (ICH) emerged as noteworthy stroke types in our study. In relative terms, ICAD accounts for a substantially greater portion of strokes in Asia in comparison to Australia [19]. Previous observational studies have shown an increased incidence of ICAD among CALD groups, including African American, Hispanic, and Asian populations, compared to Caucasians. While the aetiology of ICAD is likely multifaceted, incorporating both environmental and genetic elements, hypercholesterolemia and diabetes were identified as prominent modifiable risk factors within these demographics. However, there exists a gap in data regarding the prevalence of hypercholesterolemia and diabetes specifically among Vietnamese Asians with ICAD, and our study suggests that these risk factors play a role in its development within this population [20]. Treatment options for symptomatic ICAD are limited [21], and the disease often has a complex natural history with less favourable functional outcomes [18], higher stroke recurrence rates, and higher mortality [22,23,24]. This highlights the importance of addressing modifiable risk factors such as dyslipidaemia and diabetes, which we identified as independent predictors of ICAD among Vietnamese patients.

### 4.3. ICH

The higher incidence of ICH observed among Vietnamese patients underlines the importance of ethnicity as a significant predictor for this stroke subtype. These findings align with previous research emphasizing the prominence of ICH in Vietnam, surpassing levels observed in higher income countries [25]. Furthermore, a cohort study in Vietnam highlighted the substantial mortality associated with haemorrhagic stroke, further emphasizing the importance of risk factor control [26]. This notable prevalence of ICH in the Vietnamese population may be attributed, at least in part, to insufficiently treated hypertension, highlighting a key area for targeted intervention [25]. For example, in a Vietnamese study, dietary salt reduction was shown to have the potential for substantial population health and economic benefits [26]. Achieving salt reduction targets through community engagement in SWS (and beyond) could significantly reduce the prevalence of hypertension. In addition, the lack of awareness and adherence to hypertension management standards likely contributes to ICH risk, highlighting the need to improve awareness and standards of care in stroke prevention efforts [27].

The study findings revealed that being prescribed statins was identified as an independent predictor of intracerebral haemorrhage (ICH) in the subgroup analyses of the Vietnamese cohort. The reasons for this association are unclear but may in part be attributed to the significantly higher rate of dyslipidaemia observed among Vietnamese patients compared to the Caucasian cohort. The observed association between statin therapy and intracranial haemorrhage (ICH) is consistent with a study of a diverse patient population reporting a possible increased risk of haemorrhagic stroke in stroke patients taking statins [28], alongside a potential correlation between elevated statin dose, more pronounced reductions in LDL levels, and risk of ICH [29]. Despite the cardiovascular advantages and reduced ischemic stroke risk associated with statins, further research is needed to elucidate the risk–benefit dynamics and risk of ICH with statin use in this patient cohort.

### 4.4. Treatment and Stroke Outcome

Treatment rates and their influence on clinical outcomes represent critical aspects of stroke care. Our study revealed disparities in acute stroke intervention rates, with Vietnamese patients receiving these interventions less frequently. This is consistent with prior studies highlighting potential disparities in healthcare access among Asian American patients [30]. Asian American patients have been reported to present with more severe ischemic strokes, leading to worse functional outcomes. They also receive intravenous tissue plasminogen activator (IV tPA) less frequently than Caucasian patients [30]. Factors contributing to such disparities in treatment rates may include cultural beliefs, language barriers, limited health literacy, access to healthcare services, and socioeconomic status [31]. A recent study examining temporal trends in public awareness of stroke in diverse patient populations has indicated that elevated stroke risk factors and a significant lack of awareness of reperfusion treatment options exist in minority populations in SWS compared to a Caucasian population [31]. In the present study, patients born in Vietnam were less likely to present within the 4.5 h time window for IV tPA eligibility than their Caucasian counterparts, denying some patients the opportunity for tissue reperfusion and improved functional outcome. Addressing these disparities requires culturally sensitive healthcare delivery, public health campaigns, and increased awareness within the Vietnamese community about the importance of early stroke recognition and treatment-seeking behaviour. Promisingly, campaigns in Indigenous Australian communities have set a precedent for tailored care. For example, the Australian Stroke Alliance’s Indigenous Leadership Council is dedicated to culturally sensitive healthcare delivery and public health campaigns. Their initiatives include community engagement, providing culturally safe care pathways, timely access to care, evidence-based care, improved risk screening, and primary prevention. Through these efforts, they aim to enhance stroke care while respecting Indigenous knowledge and perspectives [32]. Many of these approaches are transferrable and have the potential to form the basis for engagement programs in Vietnamese and other communities throughout Australia.

### 4.5. Strengths and Limitations

The strengths of this study include a substantial sample size with granular data collection, allowing for comprehensive analysis. The retrospective design offers efficiency and cost-effectiveness in accessing a large data pool. Additionally, being the largest observational study of stroke in Australasia focused on Vietnamese communities, this study enriches our understanding of stroke epidemiology, identifying unique characteristics within this migratory cohort.

The limitations of this study also include the retrospective design, which may introduce selection bias as it relies on hospital records and may not capture all the relevant data. The study’s findings may not be generalizable beyond the specific region and population studied, although the methods applied are likely to be transferrable. While our study has endeavoured to account for a range of confounding variables, it is important to acknowledge the potential influence of numerous unknown factors including cultural influences, dietary habits, and healthcare-seeking behaviours. These variables may interact with ethnicity in complex ways, contributing to the observed differences in stroke profiles between Vietnamese and Caucasian patients. Further investigation into these cultural nuances and their impact on health outcomes is warranted to provide a comprehensive understanding of the disparities observed in our study. It is also important to note that while associations are identified, causality cannot be definitively established without studying the success of targeted interventions, which will form the next phase of the current study. The exclusion of patients with an unknown diagnosis may introduce bias, and there may be unmeasured confounding factors affecting the results.

## 5. Conclusions

Stroke and TIA survivors from Vietnamese communities in SWS have unique cerebrovascular disease profiles compared to Caucasians. Modifiable risk factors that independently predict ICAD in patients of Vietnamese heritage were identified in this study. These results underscore the complex interplay of cultural, genetic, and environmental factors influencing stroke risk profiles among different ethnic populations and offer valuable insights to guide future approaches for stroke prevention and care in culturally diverse communities.

## Figures and Tables

**Table 1:** Comparison of stroke characteristics and receipt of treatment between Vietnamese and Caucasian patients.

	Vietnamese (*n* = 405)	Caucasian (*n* = 390)	*p*-Value
Intracranial atherosclerotic disease (ICAD)			
IS: ICAD (with ≥50% stenosis), n (%)	100 (30.9%)	27 (6.9%)	<0.001
Symptomatic ICAD	75 (23.1%)	13 (3.3%)	<0.001
Mention of ICAD with or without stenosis	161 (39.8%)	58 (14.9%)	<0.001
Time to presentation			
Presented <4.5 h after onset of symptoms	76 (18.8%)	126 (32.3%)	<0.001
Intracranial Haemorrhage (ICH)			
ICH, n (%)	100 (24.7%)	56 (14.4%)	<0.001
Hypertensive ICH, n (%)	50 (50.0%)	19 (33.9%)	0.038
Other, n (%)	16 (16.0%)	5 (8.9%)	0.16
Unknown, n (%)	34 (34.0%)	32 (57.1%)	0.004
Ischaemic Oxfordshire Classification			
TIA, n (%)	54 (17.7%)	67 (20.1%)	0.256
LACI n (%)	54 (17.7%)	44 (13.2%)	0.07
POCI	58 (19.0%)	52 (15.6%)	0.147
PACI	119 (39.0%)	161 (48.2%)	0.012
TACI	20 (6.6%)	10 (3.0%)	0.026
Ischaemic pathophysiology (TOAST classification)			
Cardioembolic, n (%)	44 (14.4%)	85 (25.4%)	<0.001
Large vessel atheroembolic, n (%)	121 (39.7%)	96 (28.7%)	0.002
Small vessel occlusion	138 (45.2%)	141 (42.2%)	0.245
Cryptogenic, n (%) (excluding ICH)	1 (0.3%)	7 (2.1%)	0.045
Other known aetiology, n (%) (excluding ICH)	1 (0.3%)	5 (1.5%)	0.131
Treatment (excluding patients with ICH)			
ANY treatment (EVT/thrombolysis), n (%)	30 (11.8%)	86 (25.7%)	<0.001
Treatment (EVT and thrombolysis), n (%)	9 (3.5%)	25 (7.5%)	0.008
Treatment thrombolysis only	10 (3.9%)	30 (9.0%)	0.002
Treatment EVT only	11 (4.3%)	31 (9.3%)	0.003

Abbreviations: ICAD: intracranial arterial disease; ICH: intracranial haemorrhage; TIA: transient ischaemic attack; LACI: lacunar infarct; POCI: posterior circulation infarct; PACI: partial anterior circulation infarct; TACI: total anterior circulation infarct; EVT: endovascular thrombectomy.

**Table 2:** Comparison of demographics and stroke risk factors between Vietnamese and Caucasian patients.

	Vietnamese (*n* = 405)	Caucasian (*n* = 390)	*p*-Value
Patient demographics			
Age (median)	66 (25–104)	67 (31–100)	0.885
Male sex, n (%)	226 (55.8%)	220 (56.4%)	0.46
BMI (median)	23.3 (13.9–35.6)	27.6 (11.2–64)	<0.001
Vascular risk factors			
Hypertension, n (%)	339 (83.7%)	274 (70.3%)	<0.001
Two or more drugs	169 (41.7%)	93 (23.8%)	<0.001
IHD, n (%)	42 (10.4%)	79 (20.3%)	<0.001
Any AF, n (%)	67 (16.5%)	93 (23.8%)	0.007
Newly diagnosed AF, n (%)	23 (5.7%)	24 (6.2%)	0.447
Diabetes, n (%)	140 (34.6%)	100 (25.6%)	0.004
History of insulin-dependent diabetes, n (%)	32 (7.9%)	27 (6.9%)	0.348
% HbA1c (range)	6 (4.8–17)	5.7 (4.8–14.1)	=
Dyslipidaemia, n (%)	328 (81.0%)	266 (68.2%)	<0.001
On statin	306 (75.6%)	256 (65.6%)	0.001
Previous or future stroke, n (%)	93 (23.0%)	191 (49.0%)	<0.001
Total smoking, (%) (both current and ex?)	99 (24.4%)	159 (40.8%)	<0.001
Current smoker	68 (16.8%)	93 (23.8%)	0.008
Previous or current alcohol abuse (>1 drink/day)	39 (9.6%)	62 (15.9%)	0.005
Current severe alcohol abuse (>3 drinks/day)	13 (3.2%)	27 (6.9%)	0.012
CKD eGFR (range)	88 (5- >90)	85 (4- >90)	0.142
eGFR < 30, n (%)	22 (5.4%)	18 (4.6%)	0.358
eGFR < 60, n (%)	63 (15.6%)	71 (18.2%)	0.183

Abbreviations: BMI: body mass index; IHD: ischaemic heart disease; AF: atrial fibrillation; HbA1c: glycosylated haemoglobin; CKD: chronic kidney disease; eGFR: estimated glomerular filtration ratio.

## Data Availability

The data used in this study may be made available by the corresponding author on reasonable request.
